# Single-cell RNA sequencing reveals common and unique gene expression profiles in primary CD4+ T cells latently infected with HIV under different conditions

**DOI:** 10.3389/fcimb.2023.1286168

**Published:** 2023-12-12

**Authors:** Xinlian Zhang, Andrew A. Qazi, Savitha Deshmukh, Roni Lobato Ventura, Amey Mukim, Nadejda Beliakova-Bethell

**Affiliations:** ^1^ Herbert Wertheim School of Public Health and Human Longevity Science, University of California, San Diego, CA, United States; ^2^ Veterans Affairs (VA), San Diego Healthcare System and Veterans Medical Research Foundation, San Diego, CA, United States; ^3^ Department of Medicine, University of California, San Diego, CA, United States

**Keywords:** HIV latency, single-cell RNA-seq, biomarker, gene expression profiling, *in vitro* models, viral tropism, HIV latency *in vivo*

## Abstract

**Background:**

The latent HIV reservoir represents the major barrier to a cure. One curative strategy is targeting diseased cells for elimination based on biomarkers that uniquely define these cells. Single-cell RNA sequencing (scRNA-seq) has enabled the identification of gene expression profiles associated with disease at the single-cell level. Because HIV provirus in many cells during latency is not entirely silent, it became possible to determine gene expression patterns in a subset of cells latently infected with HIV.

**Objective:**

The primary objective of this study was the identification of the gene expression profiles of single latently infected CD4+ T cells using scRNA-seq. Different conditions of latency establishment were considered. The identified profiles were then explored to prioritize the identified genes for future experimental validation.

**Methods:**

To facilitate gene prioritization, three approaches were used. First, we characterized and compared the gene expression profiles of HIV latency established in different environments: in cells that encountered an activation stimulus and then returned to quiescence, and in resting cells that were infected directly via cell-to-cell viral transmission from autologous activated, productively infected cells. Second, we characterized and compared the gene expression profiles of HIV latency established with viruses of different tropisms, using an isogenic pair of CXCR4- and CCR5-tropic viruses. Lastly, we used proviral expression patterns in cells from people with HIV to more accurately define the latently infected cells *in vitro*.

**Results:**

Our analyses demonstrated that a subset of genes is expressed differentially between latently infected and uninfected cells consistently under most conditions tested, including cells from people with HIV. Our second important observation was the presence of latency signatures, associated with variable conditions when latency was established, including cellular exposure and responsiveness to a T cell receptor stimulus and the tropism of the infecting virus.

**Conclusion:**

Common signatures, specifically genes that encode proteins localized to the cell surface, should be prioritized for further testing at the protein level as biomarkers for the ability to enrich or target latently infected cells. Cell- and tropism-dependent biomarkers may need to be considered in developing targeting strategies to ensure that all the different reservoir subsets are eliminated.

## 1 Introduction

The latent HIV reservoir represents the major barrier to a cure from this virus (Chun et al., 1997; Finzi et al., 1997; Wong et al., 1997); therefore, identification of a molecular signature, and ultimately, a set of cell surface markers that can be used to eliminate all the latently infected cells is crucial. HIV reservoir is highly heterogenic. Prior studies demonstrated that HIV can persist in cells of essentially all major maturation phenotypes (Lambotte et al., 2002; Bacchus et al., 2013; Buzon et al., 2014; Jaafoura et al., 2014; Soriano-Sarabia et al., 2014; Zerbato et al., 2019) and in many functional subsets of memory cells (Tran et al., 2008; Pallikkuth et al., 2015; Sun et al., 2015; Banga et al., 2018; Dobrowolski et al., 2019). In addition, though resting CD4+ T cells were believed to be the main component of the stable latent reservoir, it is not clear whether cells that express some level of activation markers, CD69, CD25, and HLA-DR, are always fully activated and are destined to die by contraction (Falcinelli et al., 2019). Furthermore, HIV provirus can remain silent even in cells activated to proliferate (Musick et al., 2019). Several recent studies have described preferred phenotypes of cells that bear the latent HIV reservoir (Neidleman et al., 2020; Collora et al., 2022; Sun et al., 2023). Recurring observations in these studies included markers of immune checkpoint, activation and differentiation states, and phenotypes that are protective from immune-mediated killing (Neidleman et al., 2020; Collora et al., 2022; Sun et al., 2023). Despite some similarities in the signatures of reservoir cells described, there appears to be no unifying phenotypic marker that can distinguish latently infected from uninfected cells (Sun et al., 2023). Sun and colleagues proposed that the host immune activities influence the absence or presence of specific molecular signatures on latently infected cells (Sun et al., 2023).

Consistent with these observations *in vivo*, several HIV latency biomarker discovery studies performed *in vitro* reported poorly overlapping sets of differentially expressed genes (Iglesias-Ussel et al., 2013; White et al., 2016; Descours et al., 2017; Trypsteen et al., 2019). This discrepancy in the identified signatures of HIV latency is consistent with the idea that these signatures are dependent on signals from the cellular environment. Indeed, in some of these *in vitro* models, HIV latency was established in activated cells that were allowed to return to quiescence (Iglesias-Ussel et al., 2013; White et al., 2016), while other models used direct infection of resting cells (Descours et al., 2017; Trypsteen et al., 2019). Remarkably, even in studies that did perform validation of identified biomarkers using cells from people with HIV, each of the biomarkers defined only a small portion of all latently infected cells, which was evident by only modest (up to 10-fold) enrichment for the reservoir cells when antibodies against the biomarker proteins were used (Iglesias-Ussel et al., 2013; Fromentin et al., 2016; Beliakova-Bethell et al., 2022). Based on these observations, it is likely that an extended complex biomarker panel will be required to define and target the entire HIV reservoir.

Single-cell RNA sequencing (scRNA-seq) has emerged as a powerful technology to make the identification of heterogenic cellular responses and gene expression profiles associated with disease possible (Saura et al., 2023; Thomas et al., 2023). Because HIV provirus in many cells during latency is not entirely silent (Lassen et al., 2004; Wiegand et al., 2017; Yukl et al., 2018), it is possible to detect these cells by scRNA-seq. This has allowed interrogating gene expression patterns of these cells at the single-cell level, even though they represent a minority population of cells in people with HIV. However, *ex vivo* studies are limited by the need to collect an enormous amount of data to capture a few HIV-positive cells in the background of thousands of HIV-negative cells to achieve sufficient power in such gene expression comparisons. To address this problem, *in vitro* models of HIV infection can be used; however, they, too, have certain limitations. First, these models are usually short-term and do not accurately represent years-long infection of cells in people with HIV. Second, it remains challenging to prioritize the selection of promising biomarkers for testing, among the identified differentially expressed genes, when using these models.

In this study, we aimed to circumvent these limitations by conducting biomarker discovery using both the *in vitro* models and the *ex vivo* samples of CD4+ T cells from virologically suppressed people with HIV. Our primary goal was to identify gene expression profiles of latency commonly observed in cells under these various conditions: 1) cellular exposure and responsiveness to the T cell receptor (TCR) stimulus before returning to a quiescent state; 2) tropism of infecting virus; 3) study participants selected. Secondarily, we were interested in determining whether conditions of latency establishment impact the composition of the biomarker profiles. In addition to analyzing all the cells with low levels of HIV RNA expression *in vitro*, we have also defined latently infected cells more narrowly, using the HIV transcriptional patterns of CD4+ T cells *ex vivo*. The signatures identified *in vitro* using hundreds of cells were then validated *ex vivo* using dozens of cells.

Our study’s results point to common gene expression profiles associated with latent HIV infection established in different conditions. These genes should be prioritized for further testing at the protein level as biomarkers for the ability to enrich or target the latently infected cells. Remarkably, we discovered the presence of latency signatures associated with variable conditions when latency was established, including cellular exposure and responsiveness to the TCR stimulus, a subset of study participants, and the tropism of the infecting virus. All of these condition-dependent biomarkers may need to be considered in developing targeting strategies to ensure that all different reservoir subsets are eliminated.

## 2 Materials and methods

### 2.1 Primary CD4+ T cell samples

Primary CD4+ T cells from HIV seronegative donor volunteers were used to establish HIV infection *in vitro*. Cells were isolated using negative selection (StemCell Technologies, Inc., Vancouver, Canada) from the peripheral blood samples. The protocol was approved by the Institutional Review Boards of the University of California San Diego, and the Veterans Affairs San Diego Healthcare System. All participants provided written informed consent. CD4+ T cells used for *ex vivo* studies were biobanked de-identified CD4+ T cell samples from people with HIV, kindly gifted to us by Dr. Douglas Richman. Cohorts from which these samples were available were described previously (Richman et al., 2019; Bakkour et al., 2020); however, characteristics of individual samples used in this study were not available to the investigators. Of importance to the present study on latency, all participants had undetectable viral loads (less than 50 copies per milliliter of plasma) (Richman et al., 2019; Bakkour et al., 2020).

### 2.2 Viruses

The laboratory strain NL4.3 was used for the majority of the experiments. In the experiments where CXCR4- and CCR5-tropic infections were compared, the isogenic pair of NL4.3 (CXCR4-tropic) and the same virus with the JR-CSF V3 loop sequence (CCR5-tropic) (Suzuki et al., 1999) were used. The viral stocks were generated by transfecting plasmid DNA into the CEM or P4R5 T cell lines. Virus preparations were quantified for infectivity via the P4R5 MAGI blue cell assay (Day et al., 2006). Before using the CCR5-tropic virus for infection, it was incubated for 30 minutes at 4°C with the infectivity enhancement reagent (Miltenyi Biotec, Inc., Gaithersburg, MD, USA).

### 2.3 The *in vitro* models of HIV latency

In the first experiment, the two *in vitro* models of HIV latency with different mechanisms of latency establishment were used. The first model involved infection, activation of CD4+ T cells using αCD3/αCD28 antibodies, and allowing the cells to return to quiescence (the 14-day model) (Soto et al., 2022). The second model involved direct infection of the resting CD4+ T cells via co-culture with autologous productively infected cells to allow cell-to-cell viral transmission (the 10-day model) (Beliakova-Bethell et al., 2019; Soto et al., 2022). The experiments were conducted in a paired design, meaning both models were set up using cells from the same three blood donors. We conducted the scRNA-seq experiment with both models on the same day; therefore, one portion of cells designated for setting up the 10-day model of latency was viably frozen for four days.

**Figure 1:**
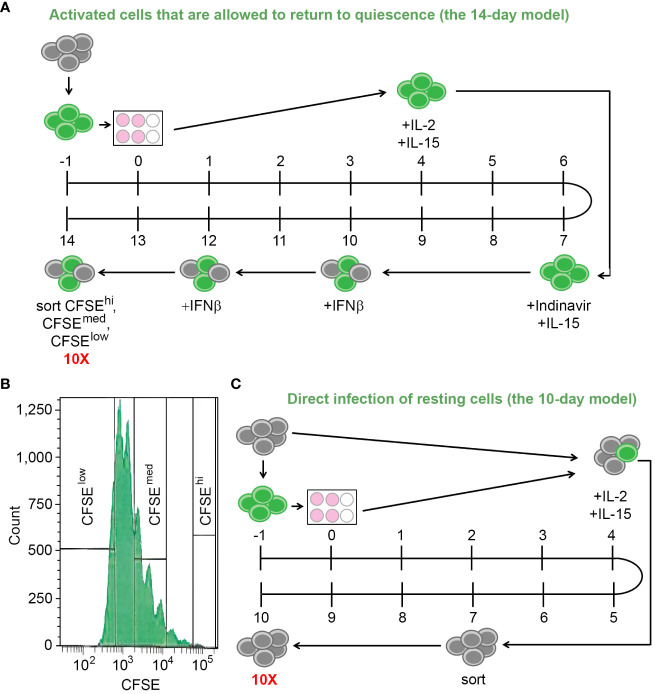
The *in vitro* models of HIV latency. **(A)** A diagram depicting the model of HIV latency established following cell activation and their return to quiescence (the 14-day model). Numbers indicate days during the model set-up; grey circles depict resting cells; green circles depict cells stained with CFSE. **(B)** Gating scheme to obtain cells with variable responsiveness to the TCR stimulus. **(C)** A diagram depicting the model of HIV latency established via cell-to-cell virus transmission from the autologous CFSE-stained, infected, activated cells (the 10-day model). Numbers indicate days during the model set-up; grey circles depict resting cells; green circles depict cells stained with CFSE. To ensure that the scRNA-seq experiment occurred on the same day, Day -1 of this model is the same day as Day 3 of the model depicted in **(A)**.

For the 14-day model, the isolated CD4+ T cells were stained with a viable dye carboxyfluorescein succinimidyl ester (CFSE, day -1). The following day, cells were infected with the wild-type HIV virus NL4.3 for 4-6 hours and then stimulated in 6-well non-tissue culture plates coated with αCD3/αCD28 antibodies (day 0). Four days later, the cells were removed from the plates and cultured in the presence of the mixture of cytokines to optimize cell proliferation and survival: IL-2, IL-15, and IFNβ, added at different times during the culture (Soto et al., 2022) (**Figure 1A**). Indinavir (1 µM) was added on Day 7 and maintained until the end of the culture. On Day 14, the cells were stained with Aqua live/dead stain (Thermo Fisher, Inc., Waltham, MA, USA), and sorted using the FACS Aria (BD Biosciences, Inc., San Jose, CA, USA) or Sony MA900 Multi-Application cell sorter (Sony, Inc., New York, NY, USA), to recover live cells with strong proliferative response to the TCR stimulus (CFSE^low^), moderate proliferative response (CFSE^med^) and no proliferative response (CFSE^high^) (**Figures 1A, B**).

For the 10-day model, the frozen cells were viably thawed and split into two portions (day -1). One portion was incubated without infection or stimulation for five days, while the other portion was stained with CFSE, infected with the wild-type NL4.3 virus, and stimulated as described above. On Day 4, the fully activated, infected, stained cells were mixed with the resting cells at a ratio of 1:4 in the presence of the cytokines IL-2 and IL-15, to allow cell-to-cell virus transmission and establishment of latent infection directly in resting cells. On Day 7, CFSE-negative resting cells were recovered by flow cytometry sorting using the FACS Aria (BD Biosciences, Inc., San Jose, CA, USA) or Sony MA900 Multi-Application (Sony, Inc., New York, NY, USA) instruments. On Day 10, the cells were stained with Aqua live/dead stain (Thermo Fisher, Inc., Waltham, MA, USA), and live cells were sorted using flow cytometry as above (**Figure 1C**).

Both the 14- and the 10-day models were extensively characterized previously (Beliakova-Bethell et al., 2019; Beliakova-Bethell et al., 2022; Soto et al., 2022). It is important to note that proviral integration frequency ranged between 2.5 and 22% for the 10-day model, and between 0.3 and 46% for the different cell subpopulations (dividing and non-dividing) in the 14-day model. Baseline RNA expression was detected for both models when quantified from bulk RNA samples isolated from a mixture of infected and uninfected cells.

The second experiment assessed the effect of viral tropism on the molecular signatures of latently infected cells. The 10-day model was used in this experiment, with freshly isolated CD4+ T cells and eFluor 670 (Thermo Fisher, Inc., Waltham, MA, USA) utilized in place of CFSE. The design was paired, meaning that cells from the same three donors were infected with CXCR4- or CCR5-tropic viruses. Of note, these three donors were different from those who participated in Experiment #1.

### 2.4 Preparation of cells for scRNA-seq

For single-cell transcriptomic profiling, the droplet-based Chromium platform developed by 10X Genomics, Inc. (Pleasanton, CA, USA) was used. Its advantages are high throughput (up to 10,000 cells per sample) and single-cell resolution. For all paired sets of experiments, scRNA-seq was conducted on the same day. Following live cell recovery using flow cytometry, CFSE^low^, CFSE^med^, and CFSE^high^ cells from the 14-day model were stained with cell hashing antibodies (Biolegend, Inc., San Diego, CA, USA) TotalSeq™-B0252, TotalSeq™-B0253, and TotalSeq™-B0254, respectively. After staining, cells were mixed back together, 10,000 from each population. For additional sample processing details, please refer to the **Supplementary Methods**. Our third, and last experiment involved biobanked CD4+ T cell aliquots from people with HIV. These cells were viably thawed, followed by Aqua live/dead staining (Thermo Fisher, Inc.) and live cell sorting. For one of the donors, two sequencing reactions were prepared (technical replicate). In all experiments, 12,000 total cells were loaded into the Chromium Controller, aiming to achieve the targeted recovery of 10,000 cells in the scRNA-Seq experiment. Reverse transcription to generate cDNA and library preparation for scRNA-seq were conducted per the manufacturer’s instructions (10X Genomics, Inc., Pleasanton, CA, USA) using v3 or v3.1 kits. Sequencing was performed at the Institute for Genomics Medicine (IGM) Genomics Center using the NovaSeq 6000 instrument (Illumina, Inc., San Diego, CA). The IGM Genomics Center provided the data as paired.fastq files.

### 2.5 ScRNA-seq read mapping and counting

Raw sequencing .fastq files were used as input into the read mapping and counting software, CellRanger v4.0 or v7.0.1, developed by 10X Genomics, Inc. (Pleasanton, CA, USA). When v7.0.1 was used, the mode to not include read mapping to gene introns was selected to directly compare the data generated earlier in time that were mapped using CellRanger v4.0. The genome reference used for mapping was the Consortium Human Reference 38 combined with the HIV genome. To maximize the chances of capturing all HIV reads, detailed information on the most abundant alternatively spliced HIV variants (Purcell and Martin, 1993) with exon coordinates was included in the genome index for CellRanger to retain potential junction reads. The output filtered_feature_bc_matrix folder, which contains the barcodes after cell-calling filtration, was used for downstream analyses in Bioconductor R. The raw and mapped data are available through the Gene Expression Omnibus (GEO) database, accession number GSE241723.

### 2.6 ScRNA-seq data pre-processing

Our data pre-processing pipeline has been recently published (Zhang et al., 2023) and was used for the samples analyzed here. Briefly, the filtered_feature_bc_matrix data generated by the CellRanger were read using the *Read10X* function in the library *Seurat* (Satija et al., 2015) in Bioconductor R. HIV unique molecular identifiers (UMIs) were removed from the gene expression matrix and added to the object metadata to ensure that cell clustering occurs based on host gene expression. Other added metadata included percent reads mapping to mitochondria genes, natural log-transformed HIV UMI, and UMI and their natural log-transformed values for all antibodies used to label cells with different proliferative responses.

A data-driven approach to exclude cells of poor quality (dead cells and multiplets) was used as described previously (Zhang et al., 2023). Of note, the multiplets were removed only from the samples generated from the 14-day *in vitro* model, where different cell hashes were used to label cells with different proliferative responses to the TCR stimulus. Removal of multiples was not feasible for the 10-day *in vitro* model or samples from people with HIV. To determine filtering thresholds, either the interquartile range (IQR) rule or the Gaussian mixture model was used based on the data (Zhang et al., 2023) (see also **Supplementary Methods**). HIV expression in individual cells was normalized to the total library size for that cell before assessing HIV expression levels in cells using the Gaussian mixture model. Histograms of HIV expression were plotted to assess the overall levels of HIV expression. It was found that a small proportion of cells had high levels of HIV expression, comparable to levels observed previously for productively infected cells (Zhang et al., 2023). These cells were therefore excluded from the analysis of differentially expressed genes between latently infected and uninfected cells.

### 2.7 Integrating replicate experiments

Data from triplicate experiments were integrated together for further analysis using the anchoring procedure (Stuart et al., 2019) in the library *Seurat*. Five integrated datasets were analyzed: Experiment #1, 14-day model; Experiment #1, 10-day model; Experiment #2 CXCR4-tropic infection; Experiment #2 CCR5-tropic infection; Experiment #3, samples from people with HIV. Before integration, gene filtering was performed to remove genes not expressed in any cells in any of the samples; data were normalized for library size and log-transformed. Two thousand genes with the most variable expression were used to identify integration anchors. Anchor expression was used to align phenotypically similar cells from the three replicate samples during the data integration process. Data were then scaled, and dimensionality reduction was first performed using principal component analysis (PCA), followed by the implementation of the t-distributed stochastic neighborhood embedding (tSNE) or uniform manifold approximation and projection (UMAP) algorithms. The integrated datasets were used for differential gene expression analyses. Integrated datasets were merged together to generate comparative plots of gene expression levels across conditions.

### 2.8 Improving the definition of latently infected cells based on HIV transcriptional profiles *ex vivo*

**Figure 2:**
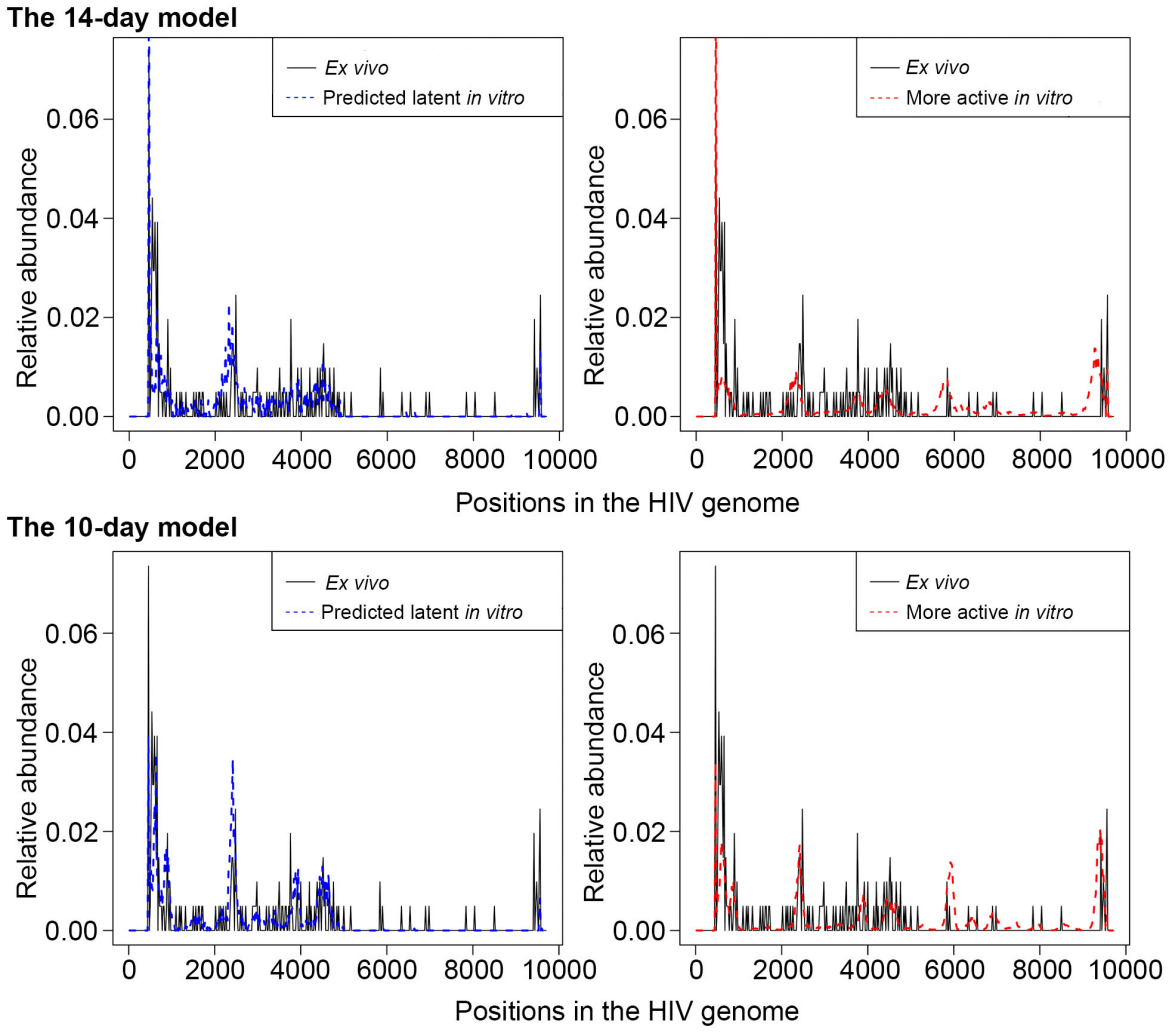
An illustration of the scaled HIV read abundance levels across the HIV genome for predicted latently infected cells (blue dash curve, left panel), with the same transcriptional profiles as in cells from people with HIV (solid black line), and cells with more active provirus (red dash curve, right panel). For both the cells from people with HIV and the predicted latently infected cells, peaks at the 3’ half of the genome are sparse and shorter than peaks at the 5’ half. For cells with more active HIV provirus, read peaks are more equally distributed and more comparable by height throughout the HIV genome. Relative abundance = (the number of reads that aligned in a specific region)/(total number of reads). Of note, relative abundance is calculated for each of the bins separately for the cells from people with HIV and cells from the *in vitro* models. Thus, comparisons between *ex vivo* and *in vitro* samples cannot be made. Rather, the heights of each peak where reads are piled up are comparable across the HIV genome within each of the groups of cells. The top panel represents cells from the 14-day model, and the bottom panel, the 10-day model of HIV latency.

Due to the HIV genome being AT-rich, the 10X platform detects HIV RNA not only at the 3’ end on the polyA tail but also in additional areas of the HIV genome with many consecutive A’s. This feature allows assessment of the HIV genome read coverage, and using it to deduce the state of HIV latency in a given cell. From the four sequenced samples represented by three people with HIV on suppressive antiretroviral therapy (ART), 71 cells expressing HIV RNA (average 3 reads per cell, range 1-35 reads per cell) were detected and used for determination of HIV transcript profiles. We parsed the .bam files from the CellRanger output using *pysam* package (Li et al., 2009; Bonfield et al., 2021) in the Python platform and extracted the starting coordinates of the mapped reads to visualize the mapped reads on the HIV genome. HIV reads mapped predominantly to the 5’ proximal half of the genome (0-5000bp), with an exceptionally high peak at the 3’ end, corresponding to the repetitive region of the long terminal repeat (LTR) in HIV transcripts. We calculated the proportion of reads falling outside the 5001bp-9526bp region, combining all HIV-positive cells from available samples from people with HIV (p0 = 0.9069). Then, for each HIV-positive cell detected *in vitro*, we compared the percentage of reads falling outside the 5001bp-9526bp region to p0. If this percentage was greater than p0, cells were labeled as “predicted latently infected cells”; provirus in other cells was considered more active, and such cells were excluded from further analyses (**Figure 2**).

### 2.9 Identification of gene expression profiles of latently infected cells

**Figure 3:**
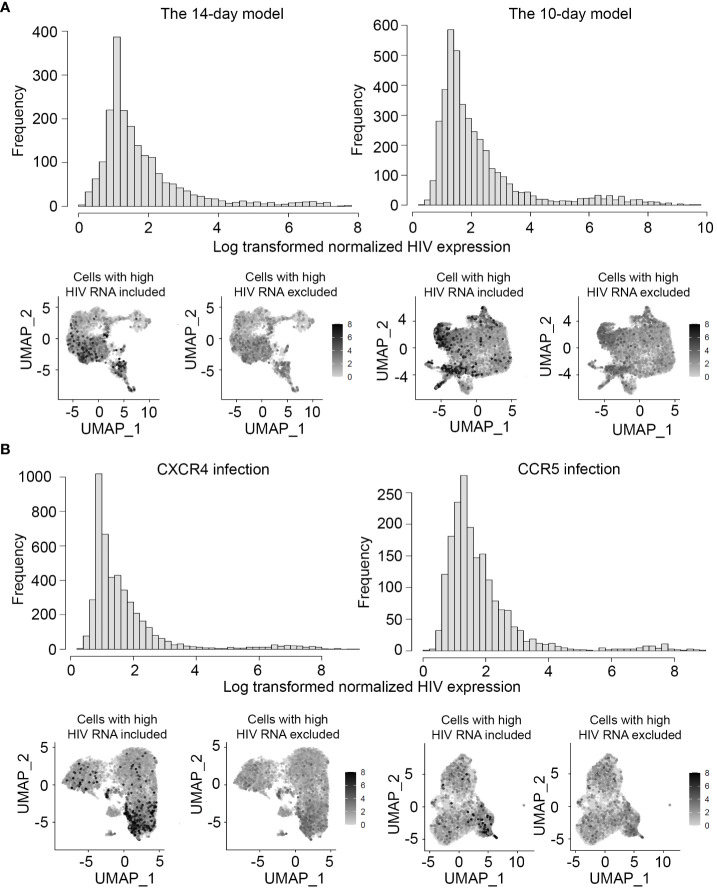
Levels of HIV RNA expression in the models of HIV latency and exclusion of cells with potential productive HIV infection. HIV expression was assessed in all sets of experiments, first by visualizing the levels of HIV RNA expression normalized to the library size using a histogram (top), and then visually on all individual cells using the *FeaturePlot* function (bottom). Feature plots are shown both before (bottom left) and after (bottom right) exclusion of cells with high levels of HIV RNA. **(A)** Experiment #1 assessing gene expression profiles of latency in the two different *in vitro* models. **(B)** Experiment #2 assessing gene expression profiles of latency established with isogenic CXCR4- and CCR5-tropic viruses.

HIV expression in individual cells was normalized to the total library size for that cell before assessing HIV expression. When HIV expression was plotted on a histogram (**Figure 3**), we noticed a minor peak with high HIV RNA levels that were comparable to the levels of HIV RNA reported previously for productively infected cells (Zhang et al., 2023). In the 14-day model, these cells may be representative of incomplete quiescence. In the 10-day model, these cells likely represent contamination of the sorted resting populations with productively infected cells. Therefore, we have used the Gaussian mixture model described above and in the **Supplementary Methods** to establish proper thresholds to exclude cells with high levels of HIV RNA from the analyses.

In the first round of analyses, we defined latently infected cells as all HIV-positive cells that remained after excluding cells with high levels of HIV RNA. In the second round of analyses, we defined “predicted latently infected cells” based on the HIV transcriptional profiles in cells from people with HIV (**Figure 2**). In both cases, differential gene expression analyses were conducted between HIV-negative cells and cells defined as latently infected with HIV. The *FindMarkers* function in the library *Seurat* was used with the default parameters. A Bonferroni-adjusted *p*-value < 0.05 was considered a significant difference. For the 14-day model, these analyses were conducted separately for CFSE^low^ (hashed with TotalSeq™-B0252), CFSE^med^ (hashed with TotalSeq™-B0253), and CFSE^high^ (hashed with TotalSeq™-B0254) cells. The identified differentially expressed genes were compared between cell subsets, between the different models, and latency established using CXCR4- and CCR5-tropic viruses.

For the identification of differentially expressed genes shared by cells in which latency was established under different conditions, the lists of genes were assessed for overlaps. We have deliberately not chosen to identify common genes by combining all the data into one large dataset, because, if all the individual samples were combined, the resulting dataset would be dominated by cells in which latency was established in a resting state. Thus, the majority of the identified markers would be expected to represent markers of resting cell infection.

### 2.10 Validation of differentially expressed genes using cells from people with HIV

The integrated dataset of samples from people with HIV was used for validation. Differential gene expression analysis was conducted between cells that had at least one HIV read and HIV-negative cells. Genes for validation were selected based on the following criteria: 1) genes were identified for cells under both definitions of latency; 2) proteins encoded by the genes localize to the plasma membrane; 3) genes were upregulated in latently infected cells. For the assessment of protein localization, the GeneCards database was used (Stelzer et al., 2016). Internally, it uses the Compartments subcellular localization database integrated from literature based on manual curation, high throughput microscopy screens, predictions from primary sequence and automatic text mining, resulting in an overall “confidence score” (scale 1-5, least to greatest confidence). Proteins with membrane localization scores of 4 or 5 were considered “localized to plasma membrane” in the present study. The *FindMarkers* function was used with selected genes as input, with all thresholds set to 0 to ensure that analysis is conducted on all selected genes, regardless of the fold change or percent of cells that express them. The *FindMarkers* function conducts multiple testing correction based on all genes detected, not the list of *a priori* selected genes. Therefore, we have conducted Bonferroni correction using the *p.adjust* function in R, which allows using *a priori* gene lists for the total number of actual tests conducted. A nominal *p*-value < 0.1 was considered a significant difference.

### 2.11 Statistical analyses

Tests of proportions were conducted to compare sets of overlapping genes identified as biomarkers of latency established in different conditions and to compare frequencies of infection with CXCR4- and CCR5-tropic viruses. The chi-squared test was used; a *p*-value < 0.05 was considered a significant difference.

## 3 Results

### 3.1 ScRNA-seq data quality assessment and cell filtering

The total number of detected cells, number of reads per cell, and number of genes detected per cell were first assessed in all experiments (**Table 1**). Data quality was assessed by identifying multiplets where possible (see Materials and Methods) and determining the percentages of reads mapping to mitochondria genes, which is indicative of cells that might be dead or dying. Multiplets and dead/dying cells were excluded from any further analyses (see **Table 1** for the number of cells analyzed in each sample).

**Table 1:** Summary of read coverage and the number of cells analyzed for all samples.

	Total cells sequenced	Mean reads per cell	Median genes per cell	Cells analyzed
Experiment #1: model comparison				
14-day model, Donor 1	2773	34198	1852	2167
14-day model, Donor 2	7551	23185	1310	4421
14-day model, Donor 3	5789	23791	1788	4634
10-day model, Donor 1	4263	33389	1561	3101
10-day model, Donor 2	8110	21406	1046	6249
10-day model, Donor 3	5593	23944	1442	5129
Experiment #2: CXCR4 *vs* CCR5				
10-day model, CXCR4, Donor 4	10041	92136	1846	6729
10-day model, CXCR4, Donor 5	5612	40532	1832	5019
10-day model, CXCR4, Donor 6	3819	61908	1865	3737
10-day model, CCR5, Donor 4	9900	93510	1926	7088
10-day model, CCR5, Donor 5	2648	100584	2200	2287
10-day model, CCR5, Donor 6	9814	26595	1417	9166
Experiment #3: cells from people with HIV				
Donor 7_1	7843	35003	1787	7245
Donor 7_2	7666	38425	1893	7070
Donor 8	7439	30790	1424	6705
Donor 9	5227	37946	1596	4603

### 3.2 Gene expression profiles of cells latently infected with HIV depend on exposure and responsiveness of CD4+ T cells to the TCR stimulus

Our prior observations indicated that active HIV infection induced differential transcriptomic remodeling in CD4+ T cells with robust, modest, and no proliferative response to the TCR stimulus (Zhang et al., 2023). Here, we aimed to determine whether gene expression profiles of latently infected cells were likewise affected by the recent exposure and responsiveness to stimulation. To this end, the two *in vitro* models of HIV latency were used. In the first model (14-day), cells were exposed to the TCR stimulus, and activated cells were allowed to return to quiescence (Soto et al., 2022). Proliferative responsiveness to the stimulus was assessed by using the CFSE dye to track the number of cell divisions. Our second model (10-day) implemented direct infection of resting CD4+ T cells via cell-to-cell virus transmission from the autologous infected, activated CD4+ T cells (Beliakova-Bethell et al., 2019; Soto et al., 2022).

Since both *in vitro* models of HIV latency represent short-term infection, we took extra precautions to evaluate the levels of HIV expression in individual cells using our scRNA-seq data. For both models, HIV expression levels (UMI) exhibited a bimodal distribution, with most cells exhibiting low levels of HIV RNA (**Figure 3A**). The cells with high levels of HIV RNA had UMI values in the same range as in our prior study of active HIV infection (Zhang et al., 2023), and these cells were, therefore, excluded from the analyses (**Figure 3A**).

**Figure 4:**
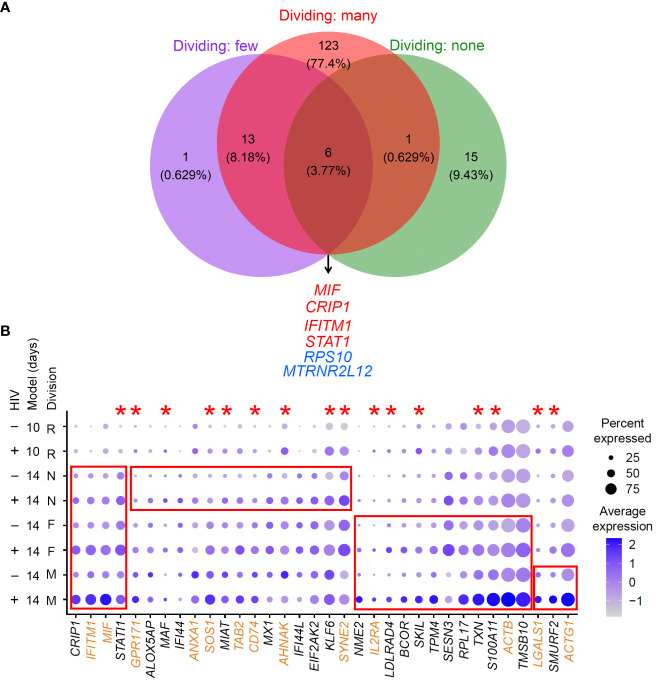
Evaluation of gene expression profiles of latency established in cells with different exposure and responsiveness to the TCR stimulus. **(A)** A Venn diagram of all significant markers for cells that were exposed to the TCR stimulus and divided many times, a few times, or remained non-dividing (the 14-day model). Overlapping genes are listed. *Red*, upregulated genes; *blue*, downregulated genes. **(B)** Expression of genes that were identified as differentially expressed in common or uniquely for different cell subsets (the 14-day model) visualized using the *DotPlot* function; their expression is also shown for the 10-day model. The size of the circle indicates the percentage of cells where each marker is expressed; the color indicates the average level of expression (log normalized scaled UMI). The red boxes emphasize cases where genes were significantly differentially expressed between HIV-negative cells and cells with low levels of HIV RNA in the 14-day model (Bonferroni-corrected *p*-value < 0.05). The red asterisks indicate genes that were also upregulated in latency established directly in resting cells via cell-to-cell viral transmission from the autologous activated productively infected cells (the 10-day model). Proteins encoded by genes highlighted in brown are localized to the plasma membrane (scores 4 or 5 in the GeneCards database). Key to the left of the dot plot shows HIV expression in cells represented in each row (- or +), model (10- or 14-day), and cell division (R, resting for the 10-day model; N, non-dividing; F, dividing a few times; M, dividing many times).

The gene expression profiles of cells that divided many times, a few times, or remained non-dividing in response to the TCR stimulus were analyzed separately. Specifically, gene expression in latently infected cells (cells with low levels of HIV RNA expression) in each group was compared to gene expression in cells with no HIV RNA from the same group. One hundred forty-three genes were differentially expressed for cells that divided many times, 20 genes for cells that divided a few times, and 22 genes for cells that remained non-dividing (**Supplementary Table 1**). Six genes were commonly modulated in latency regardless of CD4+ T cell responsiveness to the TCR stimulus (**Figure 4A**): macrophage migration inhibitory factor (*MIF*), cysteine rich protein 1 (*CRIP1*), interferon induced transmembrane protein 1 (*IFITM1*), signal transducer and activator of transcription 1 (*STAT1*), ribosomal protein S10 (*RPS10*), and MT-RNR2 like 12 (*MTRNR2L12*). The majority of the remaining genes that were identified for cells that divided a few times were also found among genes expressed differentially in cells that divided many times, but only one gene was in common between dividing and non-dividing cells (**Figure 4A**).

The most useful biomarkers for cell targeting are represented by proteins upregulated at the surface of latently infected cells. We, therefore, attempted to prioritize these markers by selecting upregulated genes and using information available in the GeneCards database (Stelzer et al., 2016) regarding the likelihood of protein association with the plasma membrane. **Figure 4B** depicts such markers identified for all cell types in common, shared by two cell types, or unique to one cell type.

Because cells not responsive to the TCR stimulus are only minimally activated (Soto et al., 2022), we next hypothesized that signatures of HIV latency established directly in resting cells would be most similar to those of non-dividing cells exposed to the TCR stimulus. To test this hypothesis, we used the 10-day model of HIV latency established via cell-to-cell virus transmission from autologous productively infected cells. Because of our paired experimental design, we were able to ensure that observed differences would not be due to biological differences between cell donors, but only due to the model used. Again, gene expression in latently infected cells (cells with low levels of HIV RNA) was compared to gene expression in cells with no HIV RNA. One hundred and five genes were identified as differentially expressed (**Supplementary Table 2**). Differentially expressed genes identified in cells exposed to the TCR stimulus were then compared to the markers identified for the resting cells. For cells that remained non-dividing after the TCR stimulus exposure, 11 out of 22 genes (50%) overlapped with the markers identified for the resting cells. For cells that divided a few times in response to the TCR stimulus, 8 of 20 (40%) genes overlapped with the markers identified for the resting cells. Finally, the overlap between genes identified for cells that divided many times and the resting cells constituted 28 of 143 (~20%) genes. The overlap between resting and non-dividing cells (*p*-value = 0.002139) or cells that divided a few times (*p*-value = 0.038) was significantly greater than the overlap between resting cells and cells that divided many times. These results are consistent with the idea that the biomarkers of cells with no or minimal proliferative response to the TCR stimulus are similar to those of latency established directly in resting cells. Genes that were significantly upregulated (Bonferroni-adjusted *p*-value < 0.05) in the latently infected cells from the 10-day model are indicated with asterisks in **Figure 4**. Overall, *STAT1* was upregulated consistently in all conditions.

### 3.3 Gene expression profiles of latently infected cells depend on the tropism of the infecting virus and the biological variation between the study participants

CXCR4- and CCR5-tropic viruses induce different signaling pathways in cells at the time of infection. For example, CCR5-tropic viruses increase the levels of cell proliferation and expression of activation markers (Locher et al., 2005) and can replicate in the absence of TCR-mediated re-stimulation (Vicenzi et al., 1999). On the other hand, induction of pathways associated with cytoskeleton reorganization and actin filament processing was unique for the CXCR4-tropic virus (Cicala et al., 2006), consistent with a reported induction of the cofilin pathway via engagement of the CXCR4 co-receptor (Yoder et al., 2008). Since we observed that different degree of responsiveness to the TCR stimulus was associated with different molecular signatures when latency was established, we hypothesized that exposure to different viruses may likewise cause variation in gene expression profiles of latently infected cells. To minimize the initial response associated with productive infection, the 10-day model of latency established directly in resting cells was used for these experiments. The paired design allowed to eliminate the variation in identified genes due to different biological replicates, and ensured that the gene expression differences would be due solely to the tropism of the infecting virus. Because participants who donated blood for experiments #1 and #2 were different, it was possible to evaluate the effect of biological variation on the biomarkers of latency identified from the two sets of three 10-day models established with CXCR4-tropic infection in the two independent experiments. Of note, in Experiment #1, cells were viably frozen for four days and thawed, while in Experiment #2, freshly isolated CD4+ T cells were used. However, we believe that differences due to variable experimental conditions are negligible because the gene expression readout was conducted after 10 days of culture.

As before, cells with high levels of HIV RNA were excluded from the analyses (**Figure 3B**). Because infection with CCR5-tropic virus is less efficient *in vitro* compared to CXCR4-tropic infection, we first evaluated the proportions of cells infected with viruses of different tropisms. For CXCR4-tropic infection, a total of 15,485 cells were analyzed, of which 10,891 did not have detectable HIV RNA, and 4,346 had low levels of RNA expression (248 cells had high levels of RNA expression and were excluded). Therefore, cells with low levels of HIV RNA represented 28.1%, and cells with high levels of HIV RNA 1.6% of all cells sequenced. For CCR5-tropic infection, a total of 18,541 cells were analyzed, of which 16,643 did not have detectable HIV RNA, 1,829 cells had low levels of HIV RNA expression (69 cells had high levels of HIV RNA expression and were excluded). Therefore, cells with low levels of HIV RNA represented 9.9%, and cells with high levels of HIV RNA 0.37% of all cells sequenced. As expected, infection with the CCR5 virus was less frequent compared to infection with the CXCR4 virus (*p*-value < 0.001). Nonetheless, a sufficient number of latently infected cells was sequenced to identify gene expression profiles of latency established with viruses of different tropisms.

**Figure 5:**
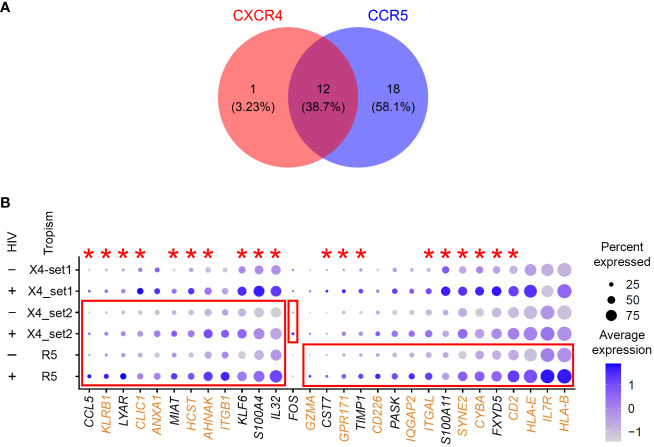
Evaluation of the gene expression profiles of latency established in cells with viruses of different tropism. **(A)** A Venn diagram of all significant markers for cells from Experiment #2 that were infected with either CXCR4- or CCR5-tropic viruses. **(B)** The 10-day model datasets from Experiment #1 (CXCR4_set1), and Experiment #2 (CXCR4_set2 and CCR5) were merged to plot the expression of genes that were identified as differentially expressed between HIV-negative and latently infected cells. The size of the circle indicates the percentage of cells where each marker is expressed; the color indicates the average level of expression (log normalized scaled UMI). The red boxes emphasize cases where genes were significantly differentially expressed between HIV-negative cells and cells with low levels of HIV RNA in the tropism experiment (Bonferroni-corrected *p*-value < 0.05). The red asterisks indicate genes that were also upregulated in latency established directly in resting cells from Experiment #1. Proteins encoded by genes highlighted in brown are localized to the plasma membrane (scores 4 or 5 in the GeneCards database). Key to the left of the dot plot shows HIV expression in cells represented in each row (- or +), and the tropism of the infecting virus (X4, CXCR4; R5, CCR5; set1 and set2 refer to Experiments #1 and 2).

Thirteen genes were significantly upregulated in cells with low levels of CXCR4-tropic HIV RNA (**Supplementary Table 3**). Thirty genes were identified as differentially expressed for CCR5-tropic infection, 28 of which were upregulated (**Supplementary Table 3**). Twelve genes were upregulated in common for CXCR4- and CCR5-tropic infection (**Figure 5A**). Of note, in this experiment, an order of magnitude fewer genes were identified for CXCR4-tropic infection, compared to the set described in Experiment #1 that assessed the effect of environmental stimuli. Therefore, there appears to be a donor effect on identifying differentially expressed genes. To test for this possibility, we have merged all three of our datasets generated with the 10-day model. We then plotted all the positive markers identified with the CCR5 tropic virus for all three datasets (**Figure 5B**). The majority of genes identified as markers for CCR5-tropic infection were also identified as markers of CXCR4-tropic infection in Experiment #1 with the 10-day model (**Figure 5B**, red asterisks), consistent with the idea that viral tropism may have a lesser contribution to signatures of latency than the results from the paired analysis initially implied.

To better understand the contribution of donor-to-donor variation and the tropism of the infecting virus to gene expression profiles of latently infected cells, we have relaxed the fold change threshold in the *FindMarkers* function from the default 0.25 to 0.1 (log scale), and repeated differential gene expression analysis for all the datasets that used the 10-day model of HIV latency. Seven hundred ninety-four genes were identified for the 10-day model set from Experiment #1, 142 genes were identified for CXCR4-tropic infection, and 278 genes for CCR5-tropic infection in Experiment #2. We then analyzed the overlap of differentially expressed genes between the two sets of different donors infected with the CXCR4-tropic virus and between the CXCR4- and CCR5-tropic infections. Keeping the CXCR4-tropic set from Experiment #2 as a constant for both comparisons, we identified 35 genes out of 790 to overlap for the donor-to-donor comparison, and 37 of 278 genes to overlap for the tropism comparison. The percentage of overlapping genes for the tropism comparison (13%) was significantly greater (*p*-value < 0.001) than the percentage of overlapping genes for the donor-to-donor comparison (4%). We, therefore, conclude that donor-to-donor variation plays a greater role in biomarker identification than the tropism of the infecting virus. Despite the sparsity of the observed substantial differences in signatures of CXCR4- and CCR5-tropic latency, we have noticed that among genes identified in our dataset, the interleukin 7 receptor (*IL7R*) was upregulated in latently infected cells for CCR5-tropic infection only (**Figure 5B**). In both sets of CCR4-tropic infection, the difference was not large, and even in the opposite direction for the Experiment #1 dataset.

### 3.4 Differentially expressed genes identified using a stricter definition of latently infected cells based on HIV transcript profiles in people with HIV

Because our *in vitro* models of HIV latency are short-term, proviral activity in cells infected with HIV is likely higher compared to that resulting from long-term infection *in vivo*. Our prior studies that utilized the same *in vitro* models demonstrated negligible production of p24 protein without reactivation (Trypsteen et al., 2019; Soto et al., 2022), consistent with the idea that the vast majority of cells are latently infected. ScRNA-seq experiments described here were consistent with these prior observations as the number of cells with high levels of HIV RNA was small. However, scRNA-seq data allowed us to observe that in a subset of cells with low levels of HIV RNA, HIV reads mapped throughout the HIV genome. These results indicate the presence of transcription events that result in the generation of full-length and potentially spliced transcripts.

Using CD4+ T cells from people with HIV, we have visualized the distribution of HIV reads, observing the predominant location of reads to the LTRs and the 5’ half of the HIV genome (**Figure 2**). The minority of reads mapping to the 3’ half of the genome were represented by 16 of 71 (22.5%) cells. This profile was used to define “predicted latently infected cells” *in vitro*, as described in Materials and Methods. Cells from both models were assessed individually and compared to the profiles observed in cells from people with HIV (**Figure 2**). In both models, we labeled any cell with the majority of reads mapping to the LTRs and the 5’ half of the HIV genome as “predicted latent” (left panels on **Figure 2**), while cells with peaks of reads in the 3’ half of the HIV genome as cells with more active HIV provirus (right panels on **Figure 2**). For differential expression analysis, only the cells that were labeled “predicted latent” were used for comparison to cells that did not have detectable HIV RNA.

When the differential expression analysis was conducted for each of our datasets, fewer genes were identified as differentially expressed (**Supplementary Table 4**), with a substantial subset of genes overlapping with those identified in the initial analyses where latency was defined more broadly as cells with low levels of HIV RNA (**Table 2**).

**Table 2:** Differentially expressed genes identified in different experiments between predicted latently infected and uninfected cells.

Cell subset	Latency defined as “low levels of HIV RNA”, total genes	Latency defined as “same transcript profiles as in people with HIV”, total genes	Latency defined as “same transcript profiles as in people with HIV”, % overlap	Overlapping genes
Dividing many times	143	43	76.7	*FAU*, *MIF*, *RPS17*, *EEF1A1*, *RPL37*, *S100A11*, *NME2*, *RPL37A*, *CRIP1*, *TMSB10*, *RBPMS*, *IFITM1*, *RPL39*, *RPS21*, *IL2RA*, *TNFRSF18*, *ACTB*, *NDFIP2*, *SNRPF*, *TOMM5*, *RPL36*, *MT-ATP8*, *RPS10*, *SAMSN*, *HNRNPA1*, *ACTG1*, *RPSA*, *RPL4*, *CAPG*, *UQCRQ*, *DUT*, *ITM2B*, *SQSTM1*
Dividing a few times	20	12	66.7	*CRIP1*, *RPS17*, *NME2*, *TPM4*, *CD52*, *IL2RA*, *BCOR*, *SESN3*
Non-dividing	22	4	50.0	*MIAT*, *ALOX5AP*
Resting	105	45	100.0	*BHLHE40*, *RGS16*, *MIAT*, *GPRIN3*, *S100A4*, *NEAT1*, *LGALS1*, *CLIC1*, *CCL5*, *RPS10*, *FXYD5*, *CD99*, *POLR2J3.1*, *REEP5*, *CD2*, *PLEC*, *PMEPA1*, *HCST*, *SOS1*, *CLDND1*, *S100A11*, *SAMSN1*, *IL2RA*, *TXN*, *H3F3B*, *CD74*, *CD7*, *MTRNR2L12*, *GBP5*, *RAC2*, *IFITM2*, *CYBA*, *GPR171*, *STAT1*, *KLF6*, *ARHGDIB*, *IRF1*, *MAF*, *PFN1*, *MT-ND6*, *IRF4*, *PPIA*, *KLRB1*, *IL32*, *HIST1H1D*
CXCR4	13	7	100.0	*AHNAK*, *S100A4*, *ITGB1*, *KLRB1*, *ANXA1*, *IL32*, *KLF6*
CCR5	30	30	83.3	*CCL5*, *HLA-B*, *IL32*, *LYAR*, *GZMA*, *IL7R*, *AHNAK*, *ITGB1*, *KLRB1*, *CD2*, *S100A4*, *TIMP1*, *CST7*, *KLF6*, *HCST*, *CD226*, *ANXA1*, *MIAT*, *GPR171*, *CLIC1*, *FXYD5*, *SYNE2*, *ITGAL*, *CYBA*, *PASK*

### 3.5 Validation of differentially expressed genes using samples from people with HIV

Next, we selected promising biomarker candidates from our discovery *in vitro* for validation using the dataset obtained using cells from people with HIV. First, we noticed that the gene expression profiles of latently infected cells differed most based on the exposure and responsiveness to the TCR stimulus, and less so based on different sets of selected study participants or the viral tropism. Therefore, we have separately evaluated the markers identified using our two different *in vitro* models of HIV latency. In all cases, we have focused on biomarkers that can be more readily moved into the testing phase: those upregulated in latency and expressed on the cell surface. Gene sets identified as differentially expressed when using the stricter definition of HIV latency were selected based on these criteria. **Table 3** summarizes these genes, their overlap between different conditions tested, up- or downregulation, and plasma membrane localization.

**Table 3:** Comparison of gene expression profiles under a stricter definition of latency established in different experimental conditions.

Model	Conditions	Genes
14-day model	Cells that divided many times	*CYP1B1*, ***MIF***, ***TNFRSF18***, *NME2*, *FAU*, ***PLPP1***, *MT-ATP8*, ***EEF1A1***, *RPS17*, *NDFIP2*, *RPL37A*, *RBPMS*, *RPL37*, *CAPG*, ***ACTB***, *RPS21*, *DCTN4*, ***IFITM1***, *STIP1*, ***TNFRSF4***, *TMSB10*, *TOMM5*, *RPL23A*, *DUT*, *SQSTM1*, ***FABP5***, *CRIP1*, *RPL39*, ***RPSA***, *SNRPF*, *HNRNPA1*, *FDFT1*, ***ITM2B***, ***ACTG1***, *UQCRQ*, *RPL36*, *YBEY*, ***ARF5***, *RPL4*
	Cells that divided a few times	*CRIP1*, *RPS17*, *NME2*, *TPM4*, ***CD52***, *BCOR*, *SESN3*
	Cells that remained non-dividing	*ALOX5AP*
Both models	Cells dividing many times, a few times, and direct infection of resting cells (CXCR4_set1)	***IL2RA***
	Cells dividing many times and direct infection of resting cells (CXCR4_set1)	*SAMSN1*, *RPS10*, *S100A11*
	Cells that remained non-dividing and direct infection of resting cells with CXCR4- (set 1) or CCR5-tropic viruses	*MIAT*
10-day model	Direct infection of resting cells, CXCR4_set1, CXCR4_set2 and CCR5	*IL32*, ***KLRB1***, ***AHNAK***, *S100A4*, *KLF6*
	Direct infection of resting cells, CXCR4_set1 and CCR5	*CCL5*, ***CD2***, ***CLIC1***, ***HCST***, ***GPR171***, *FXYD5*, ***CYBA***
	Direct infection of resting cells, CXCR4_set2 and CCR5	***ITGB1***, ***ANXA1***
	Direct infection of resting cells, CXCR4_set1	*BHLHE40*, *GPRIN3*, *LGALS1*, ***RGS16***, *NEAT1*, ***CD99***, *REEP5*, ***CLDND1***, ***SOS1***, *IRF4*, ***CD7***, *H3F3B*, ***PMEPA1***, *TXN*, ***CD74***, ***IFITM2***, *POLR2J3.1*, ***RAC2***, *STAT1*, *MTRNR2L12*, *ARHGDIB*, *PPIA*, *MAF*, *IRF1*, *GBP5*, ***PLEC***, *PFN1*, *MT-ND6*, *HIST1H1D*
	Direct infection of resting cells, CCR5	***HLA-B***, *MALAT1*, ***IL7R***, *RPS26*, *LYAR*, ***GZMA***, *TIMP1*, ***CD226***, *CST7*, *PASK*, ***SYNE2***, *LRRN3*, ***ITGAL***, *ATF7IP2*, ***LIMS1***

Red, upregulated genes; blue, downregulated genes; bold, confidence score of localization to the plasma membrane = 4; bold and underlined, confidence score of localization to the plasma membrane = 5.

The integrated dataset of samples from people with HIV was used to validate the selected genes. Because of the small sample size for HIV-positive cells in this dataset (N=71 cells), we have relaxed a definition under which we considered genes validated to nominal *p*-value < 0.1. For the 14-day model, 12 genes were tested, of which four were undetected in samples from people with HIV. Of the eight detected genes, two (25%) were validated with the relaxed criteria (**Table 4**). For the 10-day model, 27 genes were tested, of which three were undetected in samples from people with HIV. Of the 24 detected genes, eight (33.3%) were validated with the relaxed criteria (**Table 4**). Some of these genes remained significant following correction for multiple testing using the Bonferroni method (**Table 4**).

**Table 4:** Gene expression profiles of HIV latency validated in CD4+ T cells from people with HIV.

Gene symbol	Gene name	*p*-value	average log2 fold change	percent latently infected cells expressing	percent uninfected cells expressing	Bonferroni corrected *p*-value
Activated cells that returned to quiescence (14-day model)						
*ACTB*	Actin beta	0.001	0.2159	0.986	0.924	0.011
*RPSA*	Ribosomal protein SA	0.027	0.1571	0.986	0.977	0.214
Direct infection of resting cells (10-day model)						
*ITGB1*	Integrin subunit beta 1	0.000	0.4897	0.62	0.42	0.006
*GZMA*	Granzyme A	0.001	0.1020	0.127	0.045	0.033
*CLDND1*	Claudin domain containing 1	0.002	0.2870	0.479	0.311	0.049
*CD74*	CD74 molecule	0.005	0.1724	0.465	0.295	0.131
*HCST*	Hematopoietic cell signal transducer	0.015	0.2802	0.62	0.497	0.367
*PLEC*	Plectin	0.037	0.1529	0.352	0.23	0.885
*ITGAL*	Integrin subunit alpha L	0.037	0.1661	0.451	0.328	0.892
*AHNAK*	AHNAK nucleoprotein	0.072	0.2119	0.761	0.64	1.000

Red, genes identified in several, not just one, in vitro sets.

Proportions of genes validated in people with HIV were similar for genes identified following cell exposure to the TCR stimulus and directly in resting cells (25% *vs* 33.3%, *p*-value = 1). These results are consistent with the idea that the heterogeneity of cell exposure to stimuli *in vivo* likely contributes to the heterogeneity of markers expressed in latently infected cells. Furthermore, identification of the same markers upregulated in latently infected cells *in vitro* and *ex vivo* provides a framework for future experimental validation of these biomarkers for the ability to enrich for latently infected cells and to target them for elimination.

## 4 Discussion

In the recent past, the discovery of molecules that are differentially expressed in latently infected cells has been conducted using gene expression profiling methods from mixtures of latently infected and uninfected cells, or having to enrich for infected cells using reporter viruses (Iglesias-Ussel et al., 2013; White et al., 2016; Descours et al., 2017; Beliakova-Bethell et al., 2022). However, these studies had limitations such as the inability to differentiate between gene expression profiles of latency and exposure to virus, or contamination with productively infected cells. Moreover, *in vitro* studies varied by methods of latency establishment, specifically – using activated cells that were allowed to return to quiescence or direct infection of resting cells. The identified markers, not surprisingly, were different across studies, consistent with the idea that gene expression profiles of latency may depend on the history of cell exposure to various stimuli.

With the advancement of single-cell profiling technologies, it became possible to undertake biomarker discovery at the single-cell level. In the present study, we have taken advantage of the property of latent HIV provirus to not be entirely silent (Lassen et al., 2004; Wiegand et al., 2017; Yukl et al., 2018), to detect and identify cells with latent HIV infection based on low levels of HIV RNA expression *ex vivo* and *in vitro*. Overall, we identified genes expressed differentially between latently infected and uninfected cells that were reproducible across different conditions (**Figures 4**, **5**). Cells that underwent a robust proliferative response to the TCR stimulus had gene expression profiles of latency that were most distinct from other conditions (39 unique genes in **Table 3**). Biological variation between study participants was the next factor contributing to differences in identified differentially expressed genes (29 unique genes for CXCR4_set1 in **Table 3**). Finally, viral tropism had the least contribution (15 unique genes for CCR5 infection in **Table 3**). Because of long-term viral suppression *in vivo*, we also speculated that gene expression profiles of latency *ex vivo* would be more similar to those of latency established directly in resting cells *in vitro*. However, the percentage of validated genes identified for resting cells (33.3%) was only marginally and insignificantly (*p*-value = 1) higher than that for validated genes identified for cells that were exposed to the TCR stimulus (25%). It is likely that cells *in vivo* are exposed to ongoing activation stimuli due to chronic inflammation caused by HIV (Jordan et al., 2001; Hunt et al., 2008; Ishizaka et al., 2016), or possibly due to encounters with other antigens. It is therefore possible that gene expression signatures in latently infected cells may be labile and reflect recent encounters of cells in the environment. Importantly in our study, no individual upregulated gene in latently infected cells defined the entire latently infected cell population (**Figures 4**, **5**). Rather, the identified markers were expressed on fewer than 100% of latently infected cells, consistent with the idea that multiple markers will be needed to define and target the entire HIV reservoir.

**Figure 6:**
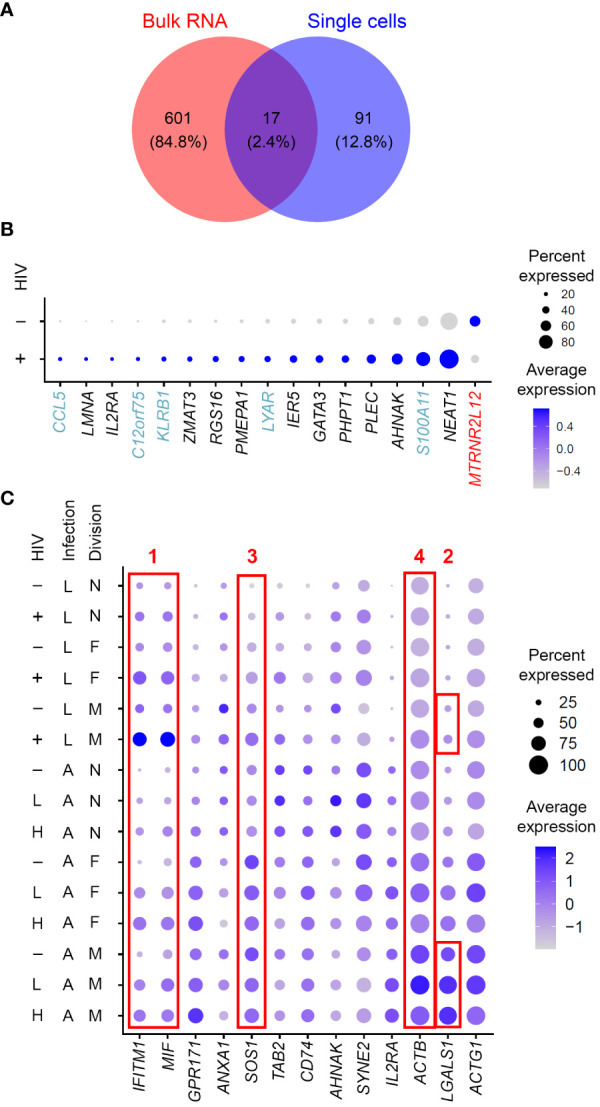
Considerations of gene expression profiles of cell exposure to the virus and productive HIV infection. **(A)** A Venn diagram of differentially expressed genes identified in a study that used bulk RNA sequencing of the 10-day model of latency compared to mock-infected cells (Trypsteen et al., 2019) and genes identified in the present study in Experiment #1 for the 10-day model. **(B)** Expression of the 17 genes found in common between the studies conducting gene expression profiling using bulk RNA (Trypsteen et al., 2019) *vs* single cells (the present study) visualized using the *DotPlot* function. The size of the circle indicates the percentage of cells where each marker is expressed; the color indicates the average level of expression (log normalized scaled UMI). *Blue*, genes that were upregulated in the present study in single latently infected cells and downregulated in the model of HIV latency relative to mock-infected cells. *Red*, genes that were downregulated in the present study but upregulated in the study by Trypsteen et al. These discrepancies may indicate the differences between reliable biomarkers of latency and signals associated with exposure to virus induced in uninfected cells. **(C)** Comparison of signatures of latent (this study) and productive (Zhang et al., 2023) HIV infection. Expression of all the genes found in both datasets that localized to the plasma membrane was visualized using the *DotPlot* function. For the latent infection dataset, cells with high levels of HIV RNA were excluded; for the productive infection dataset, cells with low and high levels of HIV RNA were visualized separately. The size of the circle indicates the percentage of cells where each marker is expressed; the color indicates the average level of expression (log normalized scaled UMI). Red boxes demonstrate four examples of optimal and suboptimal choices of biomarkers for cell targeting and enrichment strategies (see text for details). Key to the left of the dot plot shows HIV expression in cells represented in each row (- or + for the latency model and -; L, low; and H, high, for active infection), infection (L, latent; A, active) and cell division (N, non-dividing; F, dividing a few times; M, dividing many times).

An advantage of single-cell gene expression profiling studies is the ability to compare cells with and without HIV RNA from the same sample, where all cells were equally exposed to the virus (in culture or *in vivo*). This is contrary to bulk RNA sequencing experiments, where a model of HIV latency, represented by a mixture of infected and uninfected cells, is compared to control “mock-infected” cells never exposed to the virus. Conducted at the single-cell level, the present study resulted in identifying gene expression profiles representative of HIV latency, and not the exposure to virus. Prior studies profiling bulk RNA from mixtures of cells may still prove informative if mined for overlaps with scRNA-seq data. Such overlapping genes further increase the confidence of identified biomarkers. We therefore used a 10-day model dataset previously published by our group (Trypsteen et al., 2019), where all the cells exposed to the virus (both latently infected and uninfected) were compared to mock-infected controls, to assess similarities with Experiment #1 in the present study. Eleven of the 17 genes identified in both studies (**Figure 6A**), were regulated in latency in the same direction (**Figure 6B**), further validating them as biomarkers of resting cells latently infected with HIV.

Productive infection and CD4+ T cell activation are additional important factors to consider. Some gene expression profiles may be shared between productive and latent infection. This is not necessarily a reason to exclude such molecules as candidate biomarkers for developing strategies to target the latently infected cells for elimination. If a molecule is upregulated in all HIV-infected cells and these cells are targeted, then all HIV-infected cells, including both productively and latently infected cells would be eliminated. However, before selecting shared genes to develop latency eradication strategies, caution must be taken in assessing the expression of these molecules across different conditions, including activated uninfected cells. As an example of such an assessment, we have visualized the expression of upregulated plasma membrane localized biomarkers identified in the present study for the 14-day model for both the latently infected cells analyzed here and productively infected cells analyzed previously (Zhang et al., 2023) (**Figure 6C**). We note several examples of expression patterns for genes detected as markers of both productive and latent infection. First, a gene may be upregulated during productive infection and increase in expression during latency (Example #1 in **Figure 6C**). In this example, the percentage of cells that express this marker is increased in the infected cell subset, compared to the uninfected cells. Using such molecules to target latently infected cells will also destroy cells that happen to be productively infected, and only minimally eliminate the uninfected cells. A cell enrichment strategy based on such genes would most likely be more specific to the latently infected, not the productively infected cells. Second, a gene may have a higher expression on more cells during productive infection, including the uninfected cells, compared to cells that returned to quiescence (Example #2 in **Figure 6C**). Of note, this particular example is specific to cells with robust proliferative response to the TCR stimulus. In this scenario, while both latently and productively infected cells may be targeted, there is a risk of eliminating a substantial number of activated uninfected cells. Likewise, using genes like this for cell enrichment will likely result in a mixed population with a predominant population of activated productively infected, or uninfected cells. The third, perhaps worst-case scenario, is when gene expression in uninfected activated cells is higher than in productively infected cells and in resting cells, both uninfected and latently infected (Example #3 in **Figure 6C**). In this case, the activated uninfected cells would be targeted or enriched most efficiently. Fourth, the magnitude of gene expression may vary across conditions, while the percentage of cells expressing this gene stays constant (Example #4 in **Figure 6C**). This gene may not be a good candidate for a targeting strategy since such a strategy will likely tend to eliminate all cells, regardless of gene expression level. Based on these observations, we highlight the importance of investigating gene expression profiles in different conditions before selecting the most robust biomarkers for testing for their ability to enrich or target latently infected cells.

Interestingly, some previously identified markers validated in samples from people with HIV (Iglesias-Ussel et al., 2013; Beliakova-Bethell et al., 2022) were not found or confirmed in our dataset. Some explanations for this observation may include: (1) biological variation between study participants; (2) detection of expression at the RNA level in the present study *vs* testing proteins expressed on the cell surface; (3) expression of some markers on very few cells, both HIV-infected and uninfected, so that differential gene expression is underpowered. Indeed, biological variation in the present study appeared to be an important factor contributing to biomarker identification even when using the same method. This observation was also consistent with a prior study conducted in the laboratory of Dr. Nadia Roan using cytometry by time of flight (CyTOF) (Neidleman et al., 2020). The authors demonstrated that latently infected cells were more similar in longitudinal samples from the same person with HIV than cells obtained from different people (Neidleman et al., 2020). This limitation of biological variation may be mitigated by collecting data from a large number of study participants, which is usually not feasible in a single research project. Variation in molecular signatures identified at the RNA and protein levels was observed in earlier studies (White et al., 2015; Beliakova-Bethell et al., 2022), consistent with the possibility that this could be a factor here. Finally, the limitation associated with the number of cells tested particularly applies to the samples from people with HIV. In our study, a total of 25623 cells from people with HIV were analyzed, 71 of which expressed HIV RNA. Along the same lines, some of the genes that were identified here as unique signatures for some, but not other conditions of latency establishment, could be classified as “false negatives” if they are expressed in only a small number of cells. This observation highlights higher confidence of biomarkers found under more than one condition in the present study. Overall, our study has discovered novel biomarker candidates; however, independent validation of their expression in latently infected cells at the protein level in an independent set of study participants remains an important future direction.

A limitation of the present study is the inability to detect cells with integrated HIV DNA that are transcriptionally silent. Because these cells represent a minority among cells without HIV RNA, their contribution to the HIV-negative group is negligible and unlikely to affect the identification of the differentially expressed genes between latently infected and uninfected cells. What is absent is an ability to identify molecular signatures of the silent HIV reservoir. A recent study was able to sort cells with integrated HIV DNA and perform transcriptomic analysis of HIV DNA-positive cell population, compared to HIV DNA-negative cells (Clark et al., 2023). While gene expression profiles of these cells could be identified and were consistent with signatures reported in other studies (Neidleman et al., 2020; Collora et al., 2022; Sun et al., 2023), this experiment was not performed at the single-cell level, and it was not possible to attribute any observed signal to cells that do or do not express HIV RNA. In fact, the study reported detecting hundreds of RNA reads in some of their HIV DNA-positive cell aliquots (Clark et al., 2023). Moreover, the similarity of the identified signatures with the other studies that profiled cells with detectable HIV RNA [such as elevated expression of markers limiting proviral activity and enhancing cell survival (Clark et al., 2023; Sun et al., 2023)] suggests that signatures in the mixture of cells with the silent and active proviruses are likely driven by cells actively transcribing HIV. Better methods need to be developed to conduct single-cell studies to detect HIV DNA and the entire transcriptome from individual cells. Despite the current unavailability of such methods, single-cell studies of cells that express HIV RNA have merit, because the reservoir that is not entirely silent is likely responsible for viral rebound upon interruption of ART (Kearney et al., 2015), and thus constitutes the reservoir component that should be prioritized for targeting.

In conclusion, the present study has conducted a detailed characterization of gene expression profiles of HIV latency established in different conditions. Contribution of the exposure and responsiveness to the TCR stimulus, the tropism of the infecting virus, and the biological variation of the study participants were the factors that, to different degrees, contributed to variation in gene expression profiles of latently infected cells. These condition-dependent biomarkers may need to be considered in developing targeting strategies to eliminate the entire HIV reservoir. Importantly, shared differentially expressed genes were identified and assessed as potential biomarkers for reservoir enrichment and targeting. The important considerations for biomarker prioritization that we highlight include: (1) differentially expressed genes are shared between several conditions of latency establishment; (2) expression of these genes has to be specific to cells latently or productively infected with HIV, and not uninfected cells, whether they are resting or activated. Thus, the present study provides a framework for future experiments aimed at testing the candidate biomarkers and developing cell enrichment and targeting strategies.

## Data availability statement

The datasets presented in this study can be found in online repositories. The names of the repository/repositories and accession number(s) can be found in the article. The data are deposited to the Gene Expression Omnibus, accession number GSE241723.

## Ethics statement

The project was approved by the Institutional Review Boards of 1. The University of California San Diego, project 111173, approved from 08/2011 to 04/2021. 2. The Veterans Affairs San Diego Healthcare System, project H130323, approved from 06/2016 to 08/2021 and project H210040 approved 05/2021 and ongoing. The studies were conducted in accordance with the local legislation and institutional requirements. The participants provided their written informed consent to participate in this study.

## Author contributions

XZ: Data curation, Formal analysis, Resources, Software, Validation, Visualization, Writing – original draft, Writing – review & editing. AQ: Investigation, Methodology, Writing – review & editing. SD: Investigation, Methodology, Writing – review & editing. RLV: Investigation, Methodology, Writing – review & editing. AM: Investigation, Methodology, Writing – review & editing. NB-B: Conceptualization, Data curation, Formal analysis, Funding acquisition, Project administration, Resources, Supervision, Validation, Visualization, Writing – original draft, Writing – review & editing.

## References

[B1] Bacchus C. Cheret A. Avettand-Fenoël V. Nembot G. Mélard A. Blanc C.. (2013). A single HIV-1 cluster and a skewed immune homeostasis drive the early spread of HIV among resting CD4+ cell subsets within one month post-infection. PloS One 8, e64219. doi: 10.1371/journal.pone.0064219 23691172 PMC3653877

[B2] Bakkour S. Deng X. Bacchetti P. Grebe E. Montalvo L. Worlock A.. (2020). Replicate Aptima assay for quantifying residual plasma viremia in individuals on antiretroviral therapy. J. Clin. Microbiol. 58, e01400-20. doi: 10.1128/JCM.01400-20 PMC7685884 32967900

[B3] Banga R. Procopio F. A. Ruggiero A. Noto A. Ohmiti K. Cavassini M.. (2018). Blood CXCR3(+) CD4 T cells are enriched in inducible replication competent HIV in aviremic antiretroviral therapy-treated individuals. Front. Immunol. 9, 144. doi: 10.3389/fimmu.2018.00144 29459864 PMC5807378

[B4] Beliakova-Bethell N. Manousopoulou A. Deshmukh S. Mukim A. Richman D. D. Garbis S. D.. (2022). Integrated proteomics and transcriptomics analyses identify novel cell surface markers of HIV latency. Virology 573, 50–58. doi: 10.1016/j.virol.2022.06.003 35714458 PMC10427345

[B5] Beliakova-Bethell N. Mukim A. White C. H. Deshmukh S. Abewe H. Richman D. D.. (2019). Histone deacetylase inhibitors induce complex host responses that contribute to differential potencies of these compounds in HIV reactivation. J. Biol. Chem. 294, 5576–5589. doi: 10.1074/jbc.RA118.005185 30745362 PMC6462528

[B6] Bonfield J. K. Marshall J. Danecek P. Li H. Ohan V. Whitwham A.. (2021). HTSlib: C library for reading/writing high-throughput sequencing data. Gigascience 10, giab007. doi: 10.1093/gigascience/giab007 33594436 PMC7931820

[B7] Buzon M. J. Sun H. Li C. Shaw A. Seiss K. Ouyang Z.. (2014). HIV-1 persistence in CD4(+) T cells with stem cell-like properties. Nat. Med. 20, 139–142. doi: 10.1038/nm.3445 24412925 PMC3959167

[B8] Chun T.-W. Stuyver L. Mizell S. B. Ehler L. A. Mican J. Baseler M.. (1997). Presence of an inducible HIV-1 latent reservoir during highly active antiretroviral therapy. Proc. Natl. Acad. Sci. U.S.A. 94, 13193–13197. doi: 10.1073/pnas.94.24.13193 9371822 PMC24285

[B9] Cicala C. Arthos J. Martinelli E. Censoplano N. Cruz C. C. Chung E.. (2006). R5 and X4 HIV envelopes induce distinct gene expression profiles in primary peripheral blood mononuclear cells. Proc. Natl. Acad. Sci. U.S.A. 103, 3746–3751. doi: 10.1073/pnas.0511237103 16505369 PMC1533779

[B10] Clark I. C. Mudvari P. Thaploo S. Smith S. Abu-Laban M. Hamouda M.. (2023). HIV silencing and cell survival signatures in infected T cell reservoirs. Nature 614, 318–325. doi: 10.1038/s41586-022-05556-6 36599978 PMC9908556

[B11] Collora J. A. Liu R. Pinto-Santini D. Ravindra N. Ganoza C. Lama J. R.. (2022). Single-cell multiomics reveals persistence of HIV-1 in expanded cytotoxic T cell clones. Immunity 55, 1013–1031 e1017. doi: 10.1016/j.immuni.2022.03.004 35320704 PMC9203927

[B12] Day J. R. Martínez L. E. Šášik R. Hitchin D. L. Dueck M. E. Richman D. D.. (2006). A computer-based, image-analysis method to quantify HIV-1 infection in a single-cycle infectious center assay. J. Virol. Methods 137, 125–133. doi: 10.1016/j.jviromet.2006.06.019 16876264

[B13] Descours B. Petitjean G. López-Zaragoza J.-L. Bruel T. Raffel R. Psomas C.. (2017). CD32a is a marker of a CD4 T-cell HIV reservoir harbouring replication-competent proviruses. Nature 543, 564–567. doi: 10.1038/nature21710 28297712

[B14] Dobrowolski C. Valadkhan S. Graham A. C. Shukla M. Ciuffi A. Telenti A.. (2019). Entry of polarized effector cells into quiescence forces HIV latency. mBio 10, e00337–e00319. doi: 10.1128/mBio.00337-19 30914509 PMC6437053

[B15] Falcinelli S. D. Ceriani C. Margolis D. M. Archin N. M. (2019). New frontiers in measuring and characterizing the HIV reservoir. Front. Microbiol. 10, 2878. doi: 10.3389/fmicb.2019.02878 31921056 PMC6930150

[B16] Finzi D. Hermankova M. Pierson T. Carruth L. M. Buck C. Chaisson R. E.. (1997). Identification of a reservoir for HIV-1 in patients on highly active antiretroviral therapy. Science 278, 1295–1300. doi: 10.1126/science.278.5341.1295 9360927

[B17] Fromentin R. Bakeman W. Lawani M. B. Khoury G. Hartogensis W. Dafonseca S.. (2016). CD4(+) T cells expressing PD-1, TIGIT and LAG-3 contribute to HIV persistence during ART. PloS Pathog. 12, e1005761. doi: 10.1371/journal.ppat.1005761 27415008 PMC4944956

[B18] Hunt P. W. Brenchley J. Sinclair E. Mccune J. M. Roland M. Page-Shafer K.. (2008). Relationship between T cell activation and CD4+ T cell count in HIV-seropositive individuals with undetectable plasma HIV RNA levels in the absence of therapy. J. Infect. Dis. 197, 126–133. doi: 10.1086/524143 18171295 PMC3466592

[B19] Iglesias-Ussel M. Vandergeeten C. Marchionni L. Chomont N. Romerio F. (2013). High levels of CD2 expression identify HIV-1 latently infected resting memory CD4(+) T cells in virally suppressed subjects. J. Virol. 87, 9148–9158. doi: 10.1128/JVI.01297-13 23760244 PMC3754042

[B20] Ishizaka A. Sato H. Nakamura H. Koga M. Kikuchi T. Hosoya N.. (2016). Short intracellular HIV-1 transcripts as biomarkers of residual immune activation in patients on antiretroviral therapy. J. Virol. 90, 5665–5676. doi: 10.1128/JVI.03158-15 27030274 PMC4886768

[B21] Jaafoura S. De Goër De Herve M. G. Hernandez-Vargas E. A. Hendel-Chavez H. Abdoh M. Mateo M. C.. (2014). Progressive contraction of the latent HIV reservoir around a core of less-differentiated CD4(+) memory T cells. Nat. Commun. 5, 5407. doi: 10.1038/ncomms6407 25382623 PMC4241984

[B22] Jordan A. Defechereux P. Verdin E. (2001). The site of HIV-1 integration in the human genome determines basal transcriptional activity and response to Tat transactivation. EMBO J. 20, 1726–1738. doi: 10.1093/emboj/20.7.1726 11285236 PMC145503

[B23] Kearney M. F. Wiegand A. Shao W. Coffin J. M. Mellors J. W. Lederman M.. (2015). Origin of rebound plasma HIV includes cells with identical proviruses that are transcriptionally active before stopping of antiretroviral therapy. J. Virol. 90, 1369–1376. doi: 10.1128/JVI.02139-15 26581989 PMC4719635

[B24] Lambotte O. Demoustier A. De Goër M. G. Wallon C. Gasnault J. Goujard C.. (2002). Persistence of replication-competent HIV in both memory and naive CD4 T cell subsets in patients on prolonged and effective HAART. AIDS 16, 2151–2157. doi: 10.1097/00002030-200211080-00007 12409736

[B25] Lassen K. G. Bailey J. R. Siliciano R. F. (2004). Analysis of human immunodeficiency virus type 1 transcriptional elongation in resting CD4+ T cells *In vivo* . J. Virol. 78, 9105–9114. doi: 10.1128/JVI.78.17.9105-9114.2004 15308706 PMC506937

[B26] Li H. Handsaker B. Wysoker A. Fennell T. Ruan J. Homer N.. (2009). The sequence alignment/map format and SAMtools. Bioinformatics 25, 2078–2079. doi: 10.1093/bioinformatics/btp352 19505943 PMC2723002

[B27] Locher C. P. Witt S. A. Kassel R. Dowell N. L. Fujimura S. Levy J. A. (2005). Differential effects of R5 and X4 human immunodeficiency virus type 1 infection on CD4+ cell proliferation and activation. J. Gen. Virol. 86, 1171–1179. doi: 10.1099/vir.0.80674-0 15784911

[B28] Musick A. Spindler J. Boritz E. Pérez L. Crespo-Vélez D. Patro S. C.. (2019). HIV infected T cells can proliferate in *vivo* without inducing expression of the integrated provirus. Front. Microbiol. 10, 2204. doi: 10.3389/fmicb.2019.02204 31632364 PMC6781911

[B29] Neidleman J. Luo X. Frouard J. Xie G. Hsiao F. Ma T.. (2020). Phenotypic analysis of the unstimulated in *vivo* HIV CD4 T cell reservoir. eLife 9, e60933. doi: 10.7554/eLife.60933.sa2 32990219 PMC7524554

[B30] Pallikkuth S. Sharkey M. Babic D. Z. Gupta S. Stone G. W. Fischl M. A.. (2015). Peripheral T follicular helper cells are the major HIV reservoir within central memory CD4 T cells in peripheral blood from chronically HIV-infected individuals on combination antiretroviral therapy. J. Virol. 90, 2718–2728. doi: 10.1128/JVI.02883-15 26676775 PMC4810658

[B31] Purcell D. F. Martin M. A. (1993). Alternative splicing of human immunodeficiency virus type 1 mRNA modulates viral protein expression, replication, and infectivity. J. Virol. 67, 6365–6378. doi: 10.1128/jvi.67.11.6365-6378.1993 8411338 PMC238071

[B32] Richman D. D. Huang K. Lada S. M. Sun X. Jain S. Massanella M.. (2019). Replication competence of virions induced from CD4+ lymphocytes latently infected with HIV. Retrovirology 16, 4–4. doi: 10.1186/s12977-019-0466-1 30770748 PMC6377736

[B33] Satija R. Farrell J. A. Gennert D. Schier A. F. Regev A. (2015). Spatial reconstruction of single-cell gene expression. Nat. Biotechnol. 33, 495–502. doi: 10.1038/nbt.3192 25867923 PMC4430369

[B34] Saura C. A. Deprada A. Capilla-López M. D. Parra-Damas A. (2023). Revealing cell vulnerability in Alzheimer’s disease by single-cell transcriptomics. Semin. Cell Dev. Biol. 139, 73–83. doi: 10.1016/j.semcdb.2022.05.007 35623983

[B35] Soriano-Sarabia N. Bateson R. E. Dahl N. P. Crooks A. M. Kuruc J. D. Margolis D. M.. (2014). Quantitation of replication-competent HIV-1 in populations of resting CD4+ T cells. J. Virol. 88, 14070–14077. doi: 10.1128/JVI.01900-14 25253353 PMC4249150

[B36] Soto P. C. Terry V. H. Lewinski M. K. Deshmukh S. Beliakova-Bethell N. Spina C. A. (2022). HIV-1 latency is established preferentially in minimally activated and non-dividing cells during productive infection of primary CD4 T cells. PloS One 17, e0271674. doi: 10.1371/journal.pone.0271674 35895672 PMC9328514

[B37] Stelzer G. Rosen N. Plaschkes I. Zimmerman S. Twik M. Fishilevich S.. (2016). The GeneCards suite: from gene data mining to disease genome sequence analyses. Curr. Protoc. Bioinf. 54, 1.30.31–31.30.33. doi: 10.1002/cpbi.5 27322403

[B38] Stuart T. Butler A. Hoffman P. Hafemeister C. Papalexi E. Mauck W. M.. (2019). Comprehensive integration of single-cell data. Cell 177, 1888–1902.e1821. doi: 10.1016/j.cell.2019.05.031 31178118 PMC6687398

[B39] Sun H. Kim D. Li X. Kiselinova M. Ouyang Z. Vandekerckhove L.. (2015). Th1/17 polarization of CD4 T Cells supports HIV-1 persistence during antiretroviral therapy. J. Virol. 89, 11284–11293. doi: 10.1128/JVI.01595-15 26339043 PMC4645661

[B40] Sun W. Gao C. Hartana C. A. Osborn M. R. Einkauf K. B. Lian X.. (2023). Phenotypic signatures of immune selection in HIV-1 reservoir cells. Nature 614, 309–317. doi: 10.1038/s41586-022-05538-8 36599977 PMC9908552

[B41] Suzuki Y. Koyanagi Y. Tanaka Y. Murakami T. Misawa N. Maeda N.. (1999). Determinant in human immunodeficiency virus type 1 for efficient replication under cytokine-induced CD4(+) T-helper 1 (Th1)- and Th2-type conditions. J. Virol. 73, 316–324. doi: 10.1128/JVI.73.1.316-324.1999 9847335 PMC103836

[B42] Thomas D. D. Lacinski R. A. Lindsey B. A. (2023). Single-cell RNA-seq reveals intratumoral heterogeneity in osteosarcoma patients: A review. J. Bone Oncol. 39, 100475. doi: 10.1016/j.jbo.2023.100475 37034356 PMC10074210

[B43] Tran T.-A. De Goër De Herve M.-G. Hendel-Chavez H. Dembele B. Le Névot E. Abbed K.. (2008). Resting regulatory CD4 T cells: a site of HIV persistence in patients on long-term effective antiretroviral therapy. PloS One 3, e3305. doi: 10.1371/journal.pone.0003305 18827929 PMC2551739

[B44] Trypsteen W. White C. H. Mukim A. Spina C. A. De Spiegelaere W. Lefever S.. (2019). Long non-coding RNAs and latent HIV – A search for novel targets for latency reversal. PloS One 14, e0224879. doi: 10.1371/journal.pone.0224879 31710657 PMC6844474

[B45] Vicenzi E. Bordignon P. P. Biswas P. Brambilla A. Bovolenta C. Cota M.. (1999). Envelope-dependent restriction of human immunodeficiency virus type 1 spreading in CD4(+) T lymphocytes: R5 but not X4 viruses replicate in the absence of T-cell receptor restimulation. J. Virol. 73, 7515–7523. doi: 10.1128/JVI.73.9.7515-7523.1999 10438841 PMC104278

[B46] White C. H. Johnston H. E. Moesker B. Manousopoulou A. Margolis D. M. Richman D. D.. (2015). Mixed effects of suberoylanilide hydroxamic acid (SAHA) on the host transcriptome and proteome and their implications for HIV reactivation from latency. Antiviral Res. 123, 78–85. doi: 10.1016/j.antiviral.2015.09.002 26343910 PMC5606336

[B47] White C. H. Moesker B. Beliakova-Bethell N. Martins L. J. Spina C. A. Margolis D. M.. (2016). Transcriptomic analysis implicates the p53 signaling pathway in the establishment of HIV-1 latency in central memory CD4 T cells in an *In vitro* model. PloS Pathog. 12, e1006026. doi: 10.1371/journal.ppat.1006026 27898737 PMC5127598

[B48] Wiegand A. Spindler J. Hong F. F. Shao W. Cyktor J. C. Cillo A. R.. (2017). Single-cell analysis of HIV-1 transcriptional activity reveals expression of proviruses in expanded clones during ART. Proc. Natl. Acad. Sci. U.S.A. 114, E3659–E3668. doi: 10.1073/pnas.1617961114 28416661 PMC5422779

[B49] Wong J. K. Hezareh M. Günthard H. F. Havlir D. V. Ignacio C. C. Spina C. A.. (1997). Recovery of replication-competent HIV despite prolonged suppression of plasma viremia. Science 278, 1291–1295. doi: 10.1126/science.278.5341.1291 9360926

[B50] Yoder A. Yu D. Dong L. Iyer S. R. Xu X. Kelly J.. (2008). HIV envelope-CXCR4 signaling activates cofilin to overcome cortical actin restriction in resting CD4 T cells. Cell 134, 782–792. doi: 10.1016/j.cell.2008.06.036 18775311 PMC2559857

[B51] Yukl S. A. Kaiser P. Kim P. Telwatte S. Joshi S. K. Vu M.. (2018). HIV latency in isolated patient CD4+ T cells may be due to blocks in HIV transcriptional elongation, completion, and splicing. Sci. Transl. Med. 10, eaap9927. doi: 10.1126/scitranslmed.aap9927 29491188 PMC5959841

[B52] Zerbato J. M. Mcmahon D. K. Sobolewski M. D. Mellors J. W. Sluis-Cremer N. (2019). Naive CD4+ T cells harbor a large inducible reservoir of latent, replication-competent human immunodeficiency virus type 1. Clin. Infect. Dis. 69, 1919–1925. doi: 10.1093/cid/ciz108 30753360 PMC6853701

[B53] Zhang X. Deshmukh S. Mukim A. Zhang J. Beliakova-Bethell N. (2023). HIV infection elicits differential transcriptomic remodeling in CD4+ T cells with variable proliferative responses to the T cell receptor stimulus. Pathogens 12, 511. doi: 10.3390/pathogens12040511 37111397 PMC10145558

