# Supplementary material for: Single-cell RNA sequencing reveals common and unique gene expression profiles in primary CD4+ T cells latently infected with HIV under different conditions
Source: Front Cell Infect Microbiol. 2023 Dec 12;13:1286168. doi: 10.3389/fcimb.2023.1286168 (PMC10754520; doi:10.3389/fcimb.2023.1286168)
Supplement: Supplementary file 1 [file DataSheet_1.docx]

Supplementary Material

Single-cell RNA sequencing reveals common and unique gene expression profiles in primary CD4+ T cells latently infected with HIV under different conditions

Xinlian Zhang, Andrew A. Qazi, Savitha Deshmukh, Roni Lobato Ventura, Amey Mukim, Nadejda Beliakova-Bethell*****

*** Correspondence:** Nadejda Beliakova-Bethell: nbeliakovabethell@health.ucsd.edu

# Supplementary Methods

**Cell hashing to follow proliferative cell responses to the TCR stimulus**

The *in vitro* model of HIV latency, where cells were exposed to the T cell receptor (TCR) stimulus and allowed to return to quiescence (the 14-day model), used a viable dye carboxyfluorescein succinimidyl ester (CFSE) to track cell divisions. In the end of culture, when cells returned to quiescence, cells were stained with Aqua live/dead stain (Thermo Fisher, Inc.) and live cells that had divided many times (CFSE^low^), few times (CFSE^med^) and cells that had remained non-dividing (CFSE^high^) during the exposure to the TCR stimulus, were sorted using flow cytometry. Following the sort, different cell subsets were hashed with antibodies conjugated to unique oligonucleotides (Biolegend, Inc., San Diego, CA, USA): CFSE^low^ population was hashed with TotalSeq™-B0252, CFSE^med^, with TotalSeq™-B0253, and CFSE^high^, with TotalSeq™-B0254. CFSE^high^ population of non-dividing cells was also stained with antibodies to differentiate cell maturation subsets (CD45RA and CD62L) and activation state (CD69). Following incubation with these antibodies, CFSE^low^, CFSE^med^, CFSE^high^ cells were pooled together in equal proportions, 10,000 per subset, and 12,000 cells of the pool were sampled for scRNA-Seq experiments. Additionally, in 2 of the 3 replicate experiments using cells from different blood donors, an aliquot of CFSE^med^ cells was hashed with TotalSeq™-B0255 antibody and stained with isotype controls, TotalSeq™-B0090 and TotalSeq™-B0092. Five hundred of these control cells were added to the sequencing samples.

Cells from the *in vitro* model of HIV latency established directly in resting cells (the 10-day model) were stained with Aqua live/dead stain (Thermo Fisher, Inc.) in the end of culture, live cells were sorted using flow cytometry and incubated with antibodies to differentiate cell maturation subsets (CD45RA and CD62L) and activation state (CD69). Additionally, an aliquot of cells from the second replicate was hashed at the end of culture with TotalSeq™-B0255 antibody and stained with isotype controls, TotalSeq™-B0090 and TotalSeq™-B0092. The third replicate experiment was conducted in parallel with a mock-infected control, incubated in the same conditions as the latency model, but without the exposure to the virus. This mock-infected control was hashed at the end of culture with TotalSeq™-B0255 antibody and stained with isotype controls, TotalSeq™-B0090 and TotalSeq™-B0092. Cells hashed with isotype controls in replicate experiments 2 and 3 were pooled with the main experimental cells at the ratio of 1:20 before loading into the Chromium Controller.

Maturation, activation, and isotype control staining were not used for this paper. Cell hashing antibodies were used at dilution of 1:8; all other antibodies were used undiluted. All utilized antibodies are listed in **Table 1** below.

For experiments that involved CXCR4- and CCR5-tropic infection and CD4+ T cells from people with HIV, live cells were used directly, without incubation with hashing antibodies.

**Table 1. Feature barcoding and cell hashing antibodies.**

| **Antibody** | **Specificity** | **Clone** | **Isotype** | **Barcode sequence** | **cat#** |
| --- | --- | --- | --- | --- | --- |
| TotalSeq™-B0252 | Cell hash | LNH-94; 2M2 | Mouse IgG1, κ | TGATGGCCTATTGGG | 394633 |
| TotalSeq™-B0253 | Cell hash | LNH-94; 2M2 | Mouse IgG1, κ | TTCCGCCTCTCTTTG | 394635 |
| TotalSeq™-B0254 | Cell hash | LNH-94; 2M2 | Mouse IgG1, κ | AGTAAGTTCAGCGTA | 394637 |
| TotalSeq™-B0255 | Cell hash | LNH-94; 2M2 | Mouse IgG1, κ | AAGTATCGTTTCGCA | 394639 |
| TotalSeq™-B0063 | CD45RA | HI100 | Mouse IgG2b, κ | TCAATCCTTCCGCTT | 304161 |
| TotalSeq™-B0147 | CD62L | DREG-56 | Mouse IgG1, κ | GTCCCTGCAACTTGA | 304849 |
| TotalSeq™-B0146 | CD69 | FN50 | Mouse IgG1, κ | GTCTCTTGGCTTAAA | 310949 |
| TotalSeq™-B0090 | Isotype control | MOPC-21 | Mouse IgG1, κ | GCCGGACGACATTAA | 400185 |
| TotalSeq™-B0092 | Isotype control | MPC-11 | Mouse IgG2b, κ | ATATGTATCACGCGA | 400379 |

**Pre-processing of the scRNA-seq data**

***1.5 IQR rule for threshold determination***. The interquartile range (IQR) is a measure of statistical dispersion, defined as the difference between the 75^th^ percentile ($Q_{3}$) and the 25^th^ percentile ($Q_{1}$): $IQR=Q_{3}-Q_{1}$. The interquartile range is often used to find outliers in data. For example, data points that fall below $Q_{1}-1.5 * IQR$ or above $Q_{3}+1.5 * IQR$ are designated as outliers. The two parameters of our IQR function are the sequencing data to threshold and the IQR we wish to use. This function was used to identify thresholds for variables with unimodular distribution with outliers present, for example, to determine thresholds for percent of reads mapping to the mitochondria genes.

***Mixture model rule for threshold determination*.** A mixture model is one of the unsupervised learning methods for identifying the subpopulations in an overall population. In statistical terms, it models the distribution of observed measures to be a mixture distribution composed of the distributions of measures in the subpopulations. A Gaussian mixture model, in which the overall population is a combination (mixture distribution) composed of more than one different Gaussian distribution (subpopulation), is one of the most commonly used mixture models. We used the Expectation-Maximization (EM) algorithm output for mixtures of normal distributions from the R package *mixtools* ([Benaglia et al., 2009](#_ENREF_1)). The identified threshold is the value of natural log-transformed unique molecular identifiers (UMI) on which the probability of a cell coming from subpopulation on the left equals the probability of it coming from the right.

The four parameters of our mixture model thresholding function include sequencing data to threshold (e.g. natural log-transformed UMI of hashes); the number of modes/peaks/subpopulations in the data (kkt); the vector of initial values for the mean locations of modes/peaks/subpopulations (muv.init); and the random seed. The parameters kkt and muv.init are determined visually from the data; however, in some cases several combinations of kkt and muv.init are tested (e.g. when modes/peaks/subpopulations are not very well defined), in order to identify the best fit for the data. These parameters in each given case were selected based on the best model fit, indicated by the largest loglike value in the output of the threshold function and the smallest Bayesian information criterion (BIC) statistic. The code is available at <https://github.com/coralzhang/HIV_scRNA>. This model was used for thresholding of the data that had more than one mode, such as cell hashes with different antibodies.

To determine whether an IQR or a mixture model rule is more appropriate for any given dataset, histograms were plotted to determine the distribution. **Table 2** below shows all the thresholds that were identified for our different datasets.

**Table 2. Thresholds to define cells hashed with each of the antibodies and dead/dying cells based on the percentage of reads mapping to the mitochondria genes.**

|  | B0255 | pc.mtch* | B0252 | B0253 | B0254 |
| --- | --- | --- | --- | --- | --- |
| **Experiment 1: model comparison** | |  |  |  |  |
| 14-day model, Donor 1 | N/A | 11.64136 | 5.157966 | 5.266753 | 5.171255 |
| 14-day model, Donor 2 | 2.061667 | 9.264497 | 5.941782 | 5.98514 | 5.180658 |
| 14-day model, Donor 3 | 3.407188 | 10.26975 | 5.861152 | 5.951686 | 6.204517 |
| 10-day model, Donor 1 | N/A | 12.48805 | N/A | N/A | N/A |
| 10-day model, Donor 2 | 5.371121 | 8.552616 | N/A | N/A | N/A |
| 10-day model, Donor 3 | 2.860872 | 10.36576 | N/A | N/A | N/A |
| **Experiment 2: CXCR4 vs CCR5** | |  |  |  |  |
| CXCR4, Donor 4 | N/A | 8.271883 | N/A | N/A | N/A |
| CXCR4, Donor 5 | N/A | 8.27913 | N/A | N/A | N/A |
| CXCR4, Donor 6 | N/A | 8.271883 | N/A | N/A | N/A |
| CCR5, Donor 4 | N/A | 7.660129 | N/A | N/A | N/A |
| CCR5, Donor 5 | N/A | 7.992976 | N/A | N/A | N/A |
| CCR5, Donor 6 | N/A | 8.372296 | N/A | N/A | N/A |
| **Experiment 3: cells from people with HIV** | | |  |  |  |
| Donor 7, replicate 1 | N/A | 11.57254 | N/A | N/A | N/A |
| Donor 7, replicate 2 | N/A | 11.43278 | N/A | N/A | N/A |
| Donor 8 | N/A | 13.73738 | N/A | N/A | N/A |
| Donor 9 | N/A | 14.36923 | N/A | N/A | N/A |

*percent reads mapping to mitochondria genes; *N/A*, not applicable

***Identification of cells as multiplets.*** For the experiment with the 14-day model where hashing antibodies were used, it was feasible to identify cells that were multiplets. If a cell was found to be positive for more than one hash (TotalSeq™-B0252, TotalSeq™-B0253, and TotalSeq™-B0254), it was defined as a multiplet and excluded from the analyses. For the 10-day model or samples from people of HIV, all cells were analyzed and no multiplets were identified or excluded.

**References**

Benaglia, T., Chauveau, D., Hunter, D.R., and Young, D.S. (2009). mixtools: an R package for analyzing mixture models. *J Stat Softw.* 32**,** 1 - 29.
